# Distance mapping in three-dimensional virtual surgical planning in hand, wrist and forearm surgery: a tool to avoid mistakes

**DOI:** 10.1007/s11548-022-02779-w

**Published:** 2022-11-07

**Authors:** Philipp Honigmann, Marco Keller, Noémie Devaux-Voumard, Florian M. Thieringer, Damian Sutter

**Affiliations:** 1 Hand and Peripheral Nerve Surgery, Department of Orthopaedic Surgery and Traumatology, Kantonsspital Baselland (Bruderholz, Liestal, Laufen), 4101 Bruderholz, Switzerland; 2 Medical Additive Manufacturing Research Group (MAM), Department of Biomedical Engineering, University of Basel, 4123 Allschwil, Switzerland; 3 Department of Biomedical Engineering and Physics, Amsterdam Movement Sciences, Amsterdam UMC, University of Amsterdam, Amsterdam, The Netherlands; 4 Department of Oral and Cranio-Maxillofacial Surgery, University Hospital Basel, Spitalstrasse 21, 4031 Basel, Switzerland; 5 Department of Plastic and Hand Surgery, Inselspital, University Hospital, University of Bern, Bern, Switzerland

**Keywords:** Radius, Scaphoid bone, Osteotomy, Three-dimensional, Wrist, Hand, Software

## Abstract

**Purpose:**

Three-dimensional planning in corrective surgeries in the hand and wrist has become popular throughout the last 20 years. Imaging technologies and software have improved since their first description in the late 1980s. New imaging technologies, such as distance mapping (DM), improve the safety of virtual surgical planning (VSP) and help to avoid mistakes. We describe the effective use of DM in two representative and frequently performed surgical interventions (radius malunion and scaphoid pseudoarthrosis).

**Methods:**

We simulated surgical intervention in both cases using DM. Joint spaces were quantitatively and qualitatively displayed in a colour-coded fashion, which allowed the estimation of cartilage thickness and joint space congruency. These parameters are presented in the virtual surgical planning pre- and postoperatively as well as in the actual situation in our cases.

**Results:**

DM had a high impact on the VSP, especially in radius corrective osteotomy, where we changed the surgical plan due to the visualization of the planned postoperative situation. The actual postoperative situation was also documented using DM, which allowed for comparison of the VSP and the achieved postoperative situation. Both patients were successfully treated, and bone healing and clinical improvement were achieved.

**Conclusion:**

The use of colour-coded static or dynamic distance mapping is useful for virtual surgical planning of corrective osteotomies of the hand, wrist and forearm. It also allows confirmation of the correct patient treatment and assessment of the follow-up radiological documentation.

## Introduction

Virtual planning of surgical interventions and implant preparation enhances the confidence of the surgeon, reduces time in the operating room and can improve the functional outcome [1]. Since the first description of its use in corrective osteotomies of the distal radius in the late 1980s, three-dimensional (3D) virtual surgical planning (VSP) has become popular, particularly with the introduction of certified medical surgical planning software (Mimics, Materialise, Leuven, Belgium; Rhino Medical, Rhino Medical Services, Arlington, Texas, USA) [2–5].

**Fig. 1:**
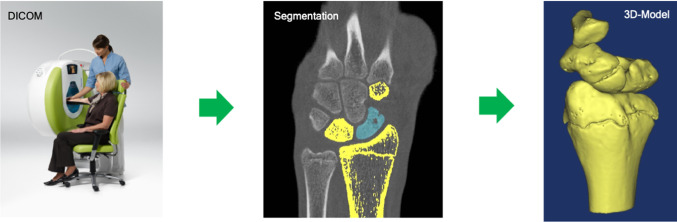
From DICOM to 3D model

Engineers as well as surgeons need to be able to rely on the accuracy of the software and tools used to plan the surgery, including aids like anatomical/pathological models, cutting guides or patient-specific implants. It is therefore mandatory to use software tools certified for medical purposes to ensure precise planning. Especially in the field of corrective osteotomies, 3D planning is particularly useful to visualize the pathology and plan the correct intervention (Fig. 1).

Distance mapping is an effective tool and can help identify planning errors leading to surgical failure and potentially poor outcomes.

Carrigan [6] developed a three-dimensional finite element model of the entire wrist to analyse load transmission pathways of the unconstrained carpus during static compressive loading.

This method was used by Rikli et al. [7] to investigate force transmission during carpal motion using a Novel® sensor introduced in the radioulnocarpal joint in vitro and in vivo.

Marai [8] introduced colour-coded distance mapping (DM) as a powerful noninvasive tool allowing the visualization of joint spaces using colour-mapped distances between cortical or cartilage layers in different positions of the wrist. This allowed an estimation of cartilage thickness. Changes in articular congruency followed by an intervention such as corrective osteotomy or degenerative changes could be visualized. In addition, Foumani [9] introduced a second criterion to define articulation areas based on the parallelism of opposing subchondral bone surfaces. He also introduced dynamic distance mapping of the radiocarpal joint based on wrist joint motion patterns in vivo, acquired by a four-dimensional X-ray imaging system that allowed dynamic assessment of bone-contact areas in different positions of the wrist [10].

Four-dimensional computed tomography (4D-CT) was introduced to the field of carpal kinematics first in cadavers and later in vivo [11, 12]. Studies in this field used distance mapping to illustrate distances between the scaphoid and the lunate [13, 14]. Recently, Robinson et al. used colour-coded distance mapping as joint congruency maps of the radioscaphoid and scapholunate joint in extreme radial and ulnar deviation to illustrate the contact areas in the joint during motion using 4D-CT [15].

We describe the use of distance mapping in surgical planning for corrective osteotomies of the distal radius and for scaphoid reconstructions in cases of nonunion using certified software in daily practice.

## Methods

### Image acquisition

We routinely use cone beam computed tomography (CBCT) to assess hand and wrist pathologies [16]. However, CBCT has a limited field of view of 13 × 16 cm and is therefore not suitable to scan complete forearms, as required in our first case. We therefore used multisliced or multidetector computed tomography (MSCT/MDCT) to obtain DICOM (Digital Imaging and Communications in Medicine) images in this case. For our second case, we used DICOM images acquired using CBCT.

### Scanning time and radiation exposure

The imaging duration for CBCT is 7.6 min ± 3.1 min for CBCT and 10.9 min ± 1.9 min for MDCT. The radiation dose for an extremity CBCT scan is 0.04 mSv ± 0.02 mSv and 0.13 mSv ± 0.07 mSv for MDCT [17].

### 3D modelling

Complete wrists were segmented into individual bone components using Disior Bonelogic 2.0 (Disior, Helsinki, Finland). The segmentation process is semiautomatic, requiring only a single mouse click per bone from the operator to correctly identify and label the bones. All subsequent steps are fully automated. Modelled cortical interfaces are represented as triangular surface meshes. The user can adjust the threshold for segmentation and repeat the modelling process.

### Distance mapping

Bonelogic software generates distance maps automatically during segmentation according to the abovementioned techniques of Marai and Foumani [8, 9]. The software computes the interbone distances between the segmented triangular meshes by scanning perpendicularly from the bone surface until an adjacent bone is reached, if any exists. This process is repeated vertex by vertex and bone by bone until all distance maps are generated. Maps are visualized by windowing the millimetre values between limits presented in accompanying colour bars. We chose to display the distance from 0 to 5 mm to enlarge the colour map area for better visualization, especially for the distances of the distal radio-ulnar joint in the first case.

We chose a colour-code from dark red (= 0 mm distance) to light blue (≥ 5 mm distance). This colour-map allows visualisation of the joint space thickness and the position of the bones of the joint. A homogeneous and centred distribution of a colour area in a joint surface corresponds to a good and centred position of the adjacent joint surfaces, whereas a coloured area outside of the joint centre indicates a malposition of the corresponded joint surface.

The data from the following cases were generated by Bonelogic and exported to MATLAB (MathWorks, Natick, MA, US) to produce further visualizations (Figs. 6, 9).

### 3D Printing

The guides used for both cases were 3D printed using biocompatible material printed with certified multi and polyjet printers.

## Cases

Static distance mapping was used in the following two cases for preoperative virtual surgical planning to verify our approach and postoperatively for quality control assessments.

### Case 1: corrective osteotomy of the forearm

**Fig. 2:**
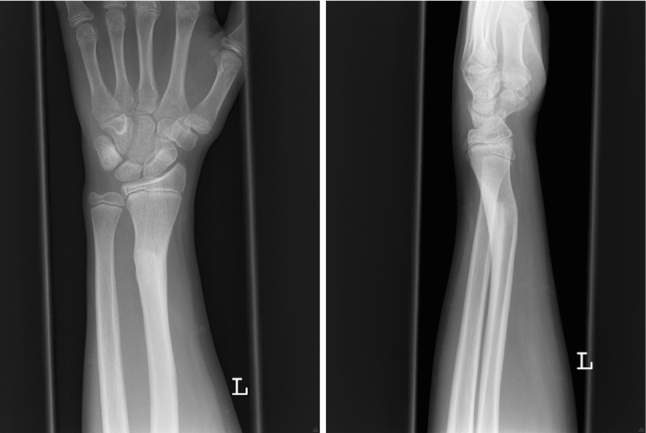
Malunited distal radius fracture dp and lat (6 months after trauma)

**Fig. 3:**
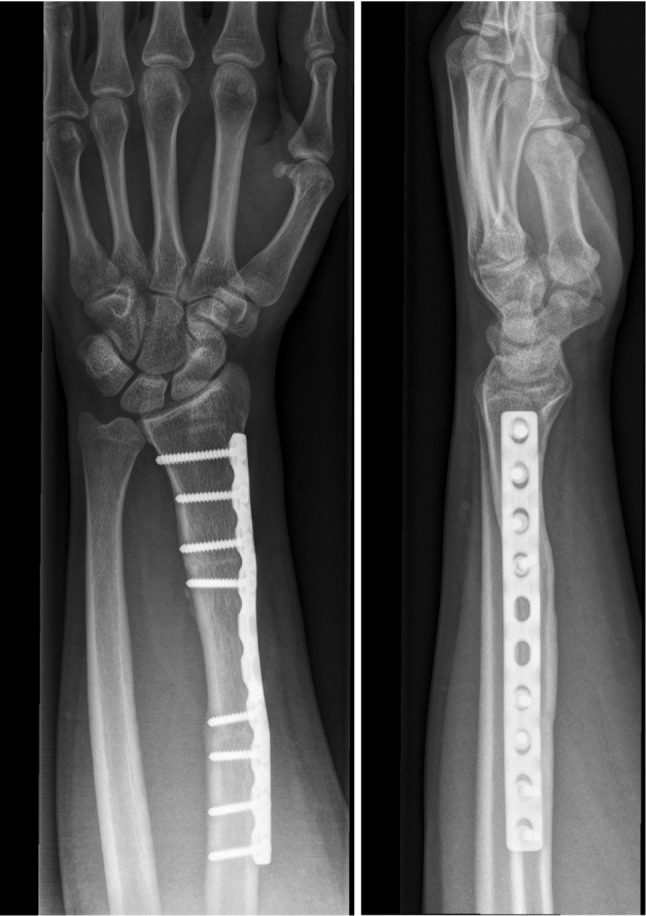
Six months after corrective osteotomy dp and lat

A 28-year-old patient sustained a Galeazzi-like distal radius fracture at the age of 14, and it was treated nonoperatively (Fig. 2). Due to residual pain and impaired range of motion, a corrective osteotomy of the radius was carried out at the age of seventeen, which healed uneventfully (Fig. 3). At the age of 27, the patient complained again about ulnar-side wrist pain with impaired supination (mechanical blockade at 75° active supination) and painful (Visual Analogue Scale—VAS 5) pronation to a maximum of 75° compared to the healthy contralateral side with 90° P/S.

**Fig. 4:**
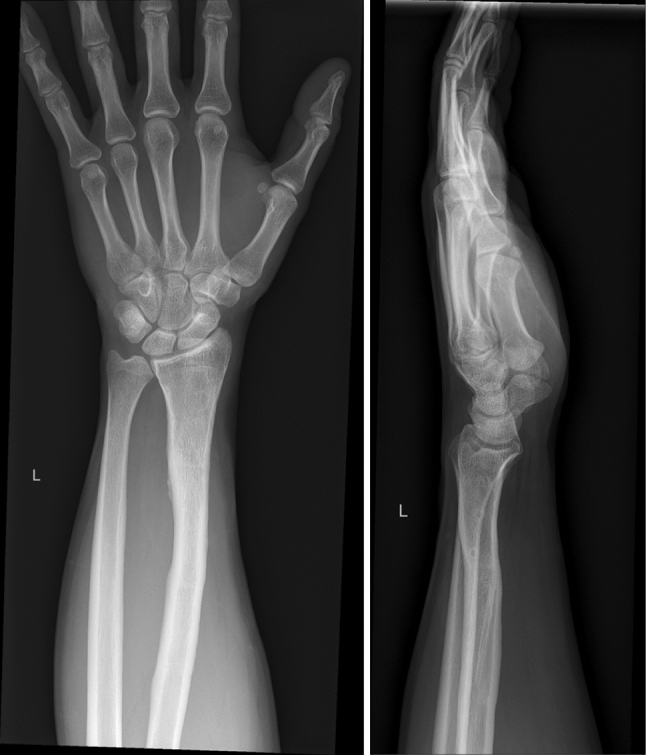
Conventional X-rays at 27 years dp and lat

Conventional X-rays revealed an ulna-neutral variant (Fig. 4). Clinically, a dynamic ulno-carpal impaction was diagnosed.

**Fig. 5:**
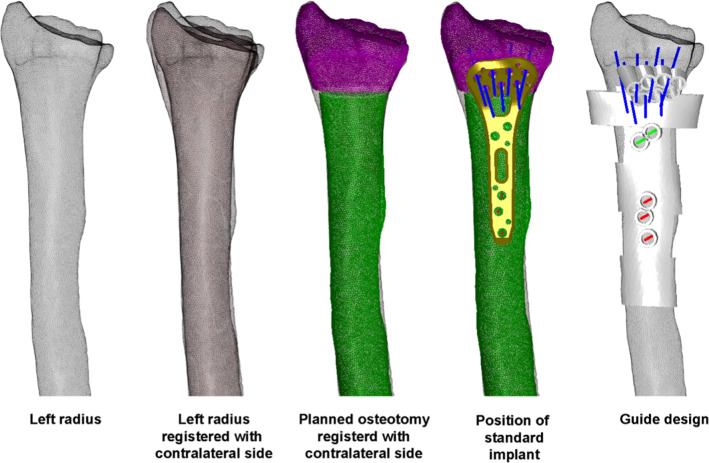
Initial virtual surgical planning. Top: registration process and planned osteotomy (purple: distal part; green: proximal part). Bottom: placement of the standard osteosynthesis plate and design of the surgical guide

Following 3D analysis based on the mirrored contralateral side, we planned a derotational osteotomy of approximately 15° of the distal radius using additively manufactured surgical guides and a standard distal radius plate (Fig. 5).

**Fig. 6:**
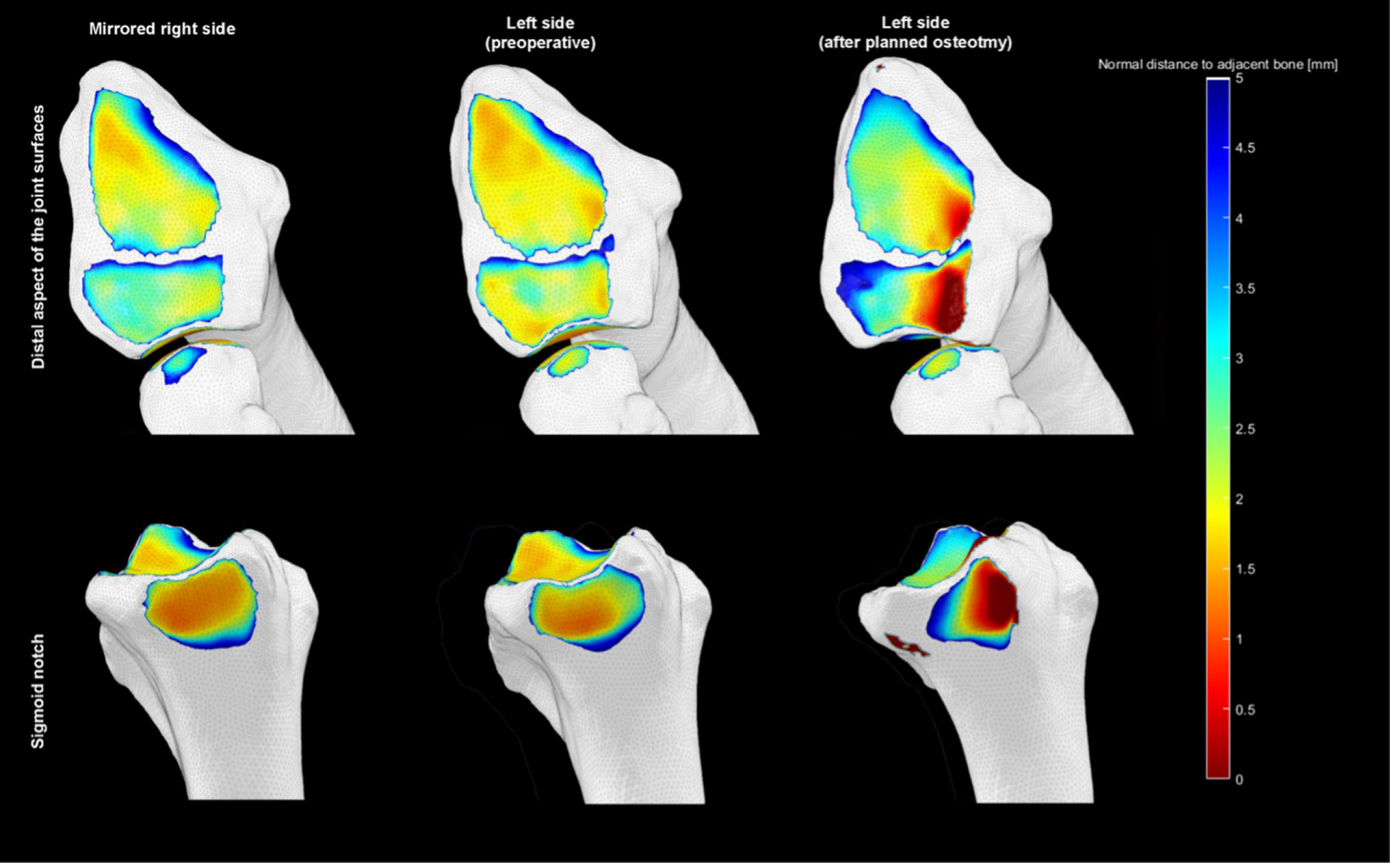
Simulation of the postoperative situation using distance mapping (dark red = 0 mm and light blue ≥ 5 mm distance). The dark red area indicates contact of the bones (ulna in the dorsal sigmoid notch and lunate in lunate fossa) which corresponds to a dorsal malposition of the ulna and lunate

The simulation of the postoperative situation using distance mapping revealed that the planned procedure would lead to an overload of the dorsal aspect of the sigmoid notch (Fig. 6).

**Fig. 7:**
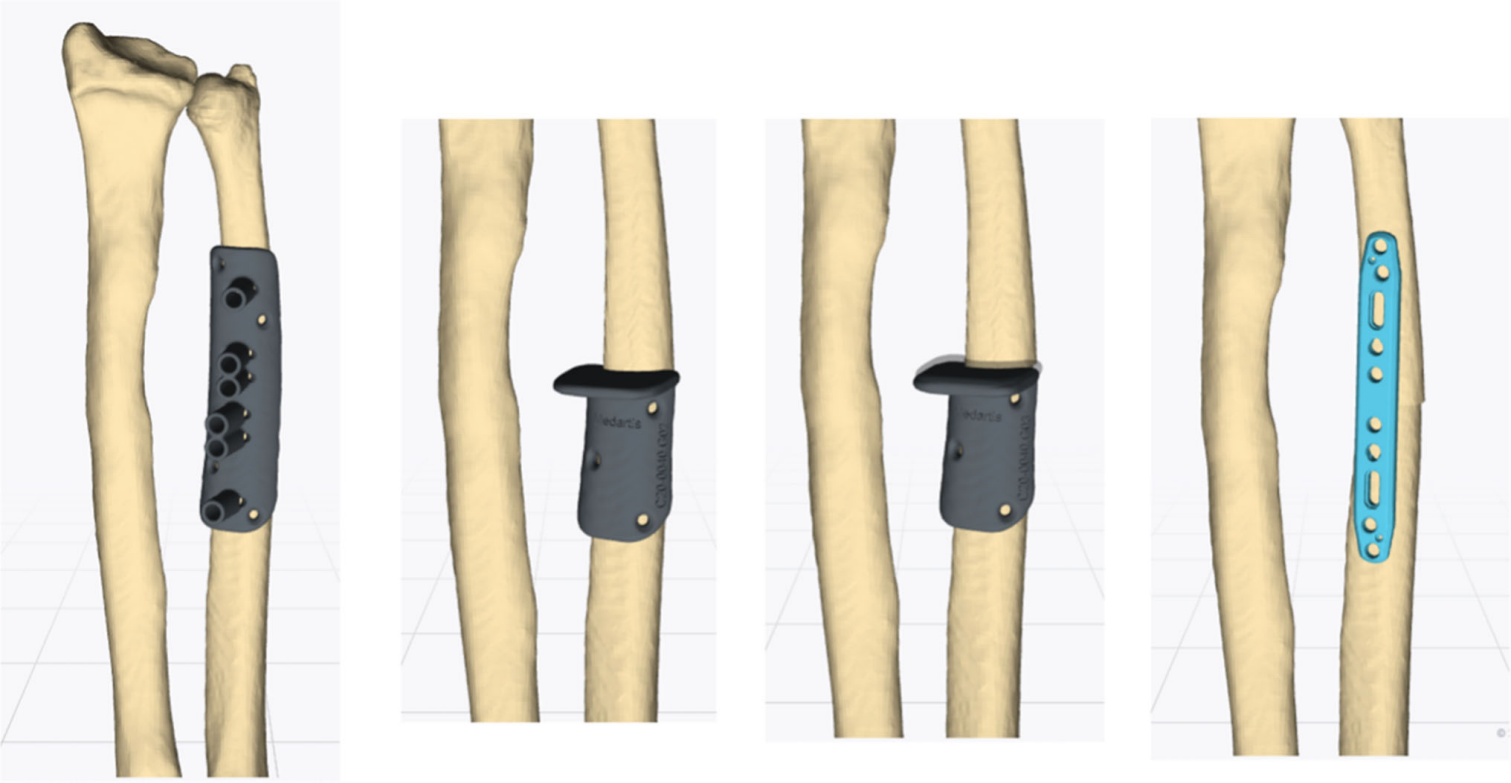
Virtual surgical planning of the derotational and shortening osteotomy of the ulna

**Fig. 8:**
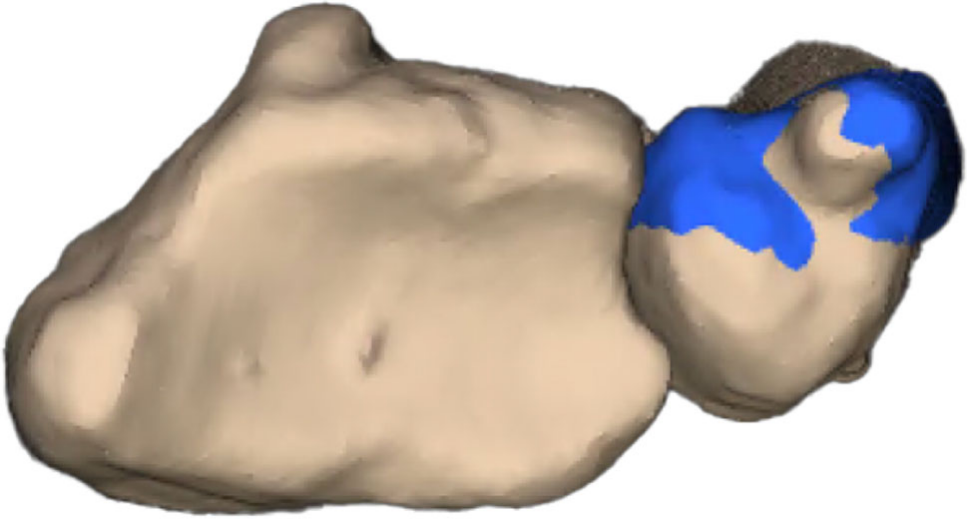
Planned position of the ulna (blue)

Based on our simulation, we changed the surgical plan to a derotational osteotomy of the ulna of approximately 15° combined with ulnar shortening using intraoperative guides and a standard ulna locking plate of 2.8 mm (Medartis AG, Basel, Switzerland) (Figs. 7, 8).

**Fig. 9:**
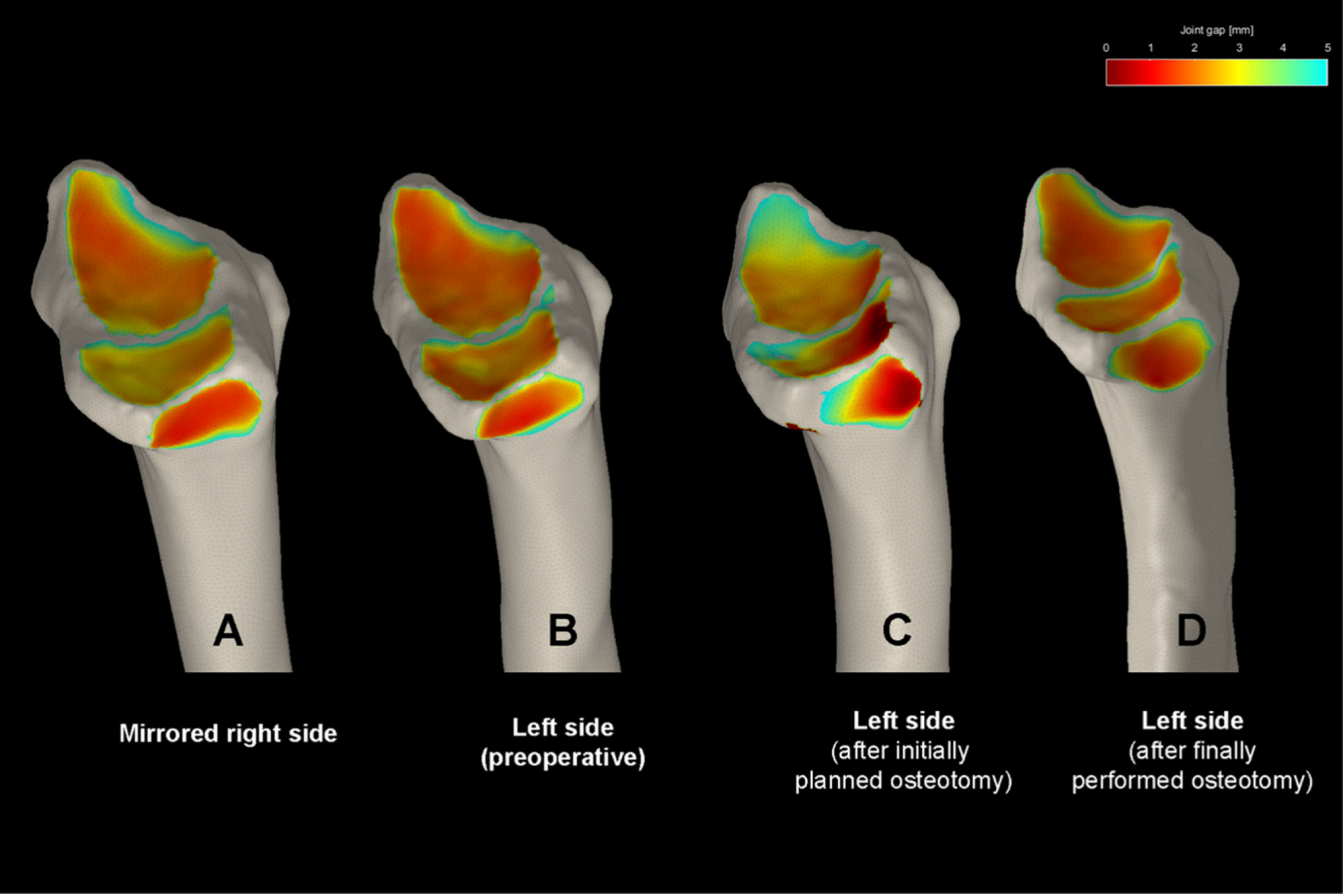
Analysis pre- and postoperatively. **A** mirrored right side as reference for virtual surgical planning. **B** symptomatic left side (preoperative). **C** left side after virtually planned situation with uncentred (dorsally) contact area of the ulna in sigmoid notch as well as a dorsal translation of the lunate in lunate fossa (dark red areas). **D** final postoperative situation on the right which shows a centred position of the ulna and a homogenous distribution of the orange area in the lunate and scaphoid fossa of the radius

The postoperative 3D analysis after 6 weeks showed an ulna-negative variant and centred position of the ulna within the sigmoid notch, as explained in Fig. 9. Clinical and radiological follow-up of the patient was performed 6 weeks, 3, 6 and 12 months postoperatively. The patient demonstrated good clinical function and regained pain-free (VAS 0) and nearly full supination of 85°.

### Case 2: scaphoid reconstruction in a case of nonunion

**Fig. 10:**
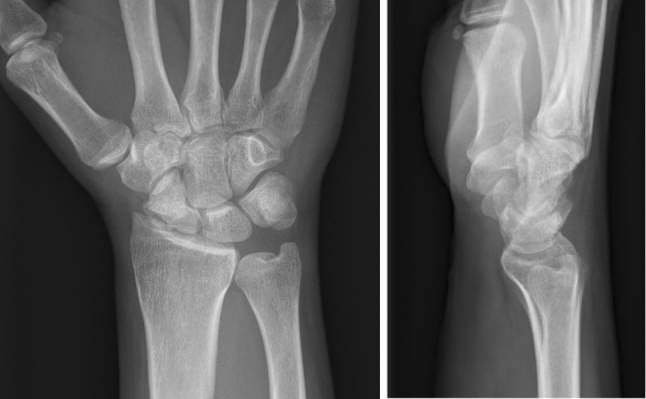
Preoperative X-rays (dorsopalmar and lateral)

A 47-year-old right-handed patient presented with a scaphoid nonunion. The X-ray showed a scaphoid-nonunion advanced collapse (SNAC) type I with degenerative articular changes of the radius styloid (Fig. 10). The scapholunate angle of 70° confirmed the dorsal-intercalated segment instability (DISI) of the lunate as seen in the X-rays of the wrist.

**Fig. 11:**
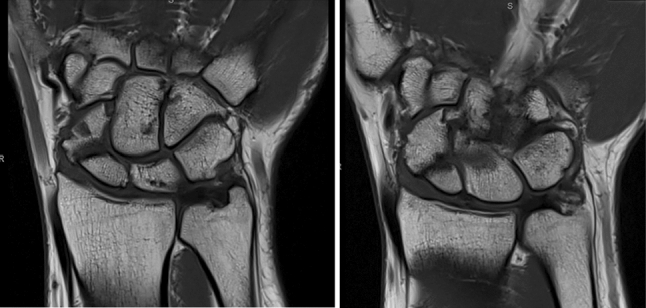
Preoperative MRI scan (coronar T1)

**Fig. 12:**
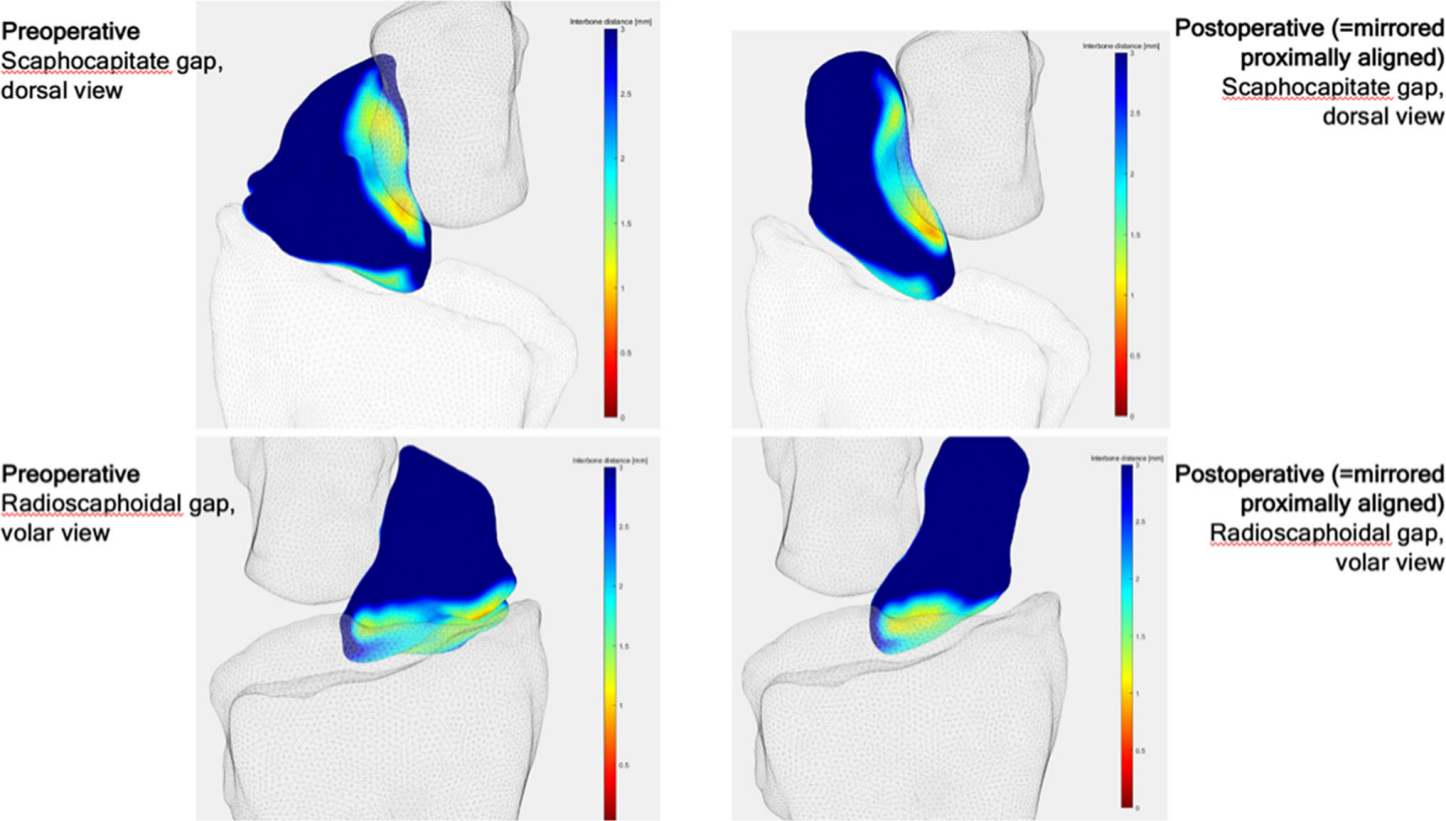
Preoperative planning using distance mapping and the mirrored opposite site

The MRI showed good vascularization of the proximal scaphoid pole with no signs of avascular necrosis (Fig. 11). DICOM images from CBCT scans were obtained from the affected and healthy wrist. Three-dimensional models using the acquired DICOM images were created using certified software (Bonelogic 2.0; DISIOR Ltd, Helsinki, Finland) to plan the reconstruction based on the mirrored contralateral side using distance mapping (Fig. 12).

**Fig. 13:**
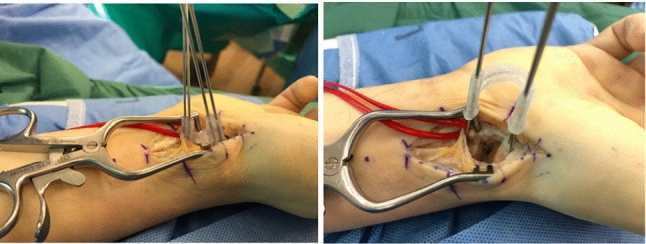
Intraoperative situation with the 3D-printed guides in place (left: guide to place K-wires; right: reposition guide to maintain parallel alignment of the K-wires)

We used additively manufactured surgical guides to place Kirschner (K)-wires according to the method first described by Haefeli et al. [18]. The planned repositioning of the scaphoid according to the mirrored contralateral side was achieved by parallelizing the inserted K-wires. A second 3D printed guide was used to hold the reposition in place and allowed for good access to the nonunion site for resection and interposition of a nonvascularized iliac-crest bone graft (Fig. 13).

**Fig. 14:**
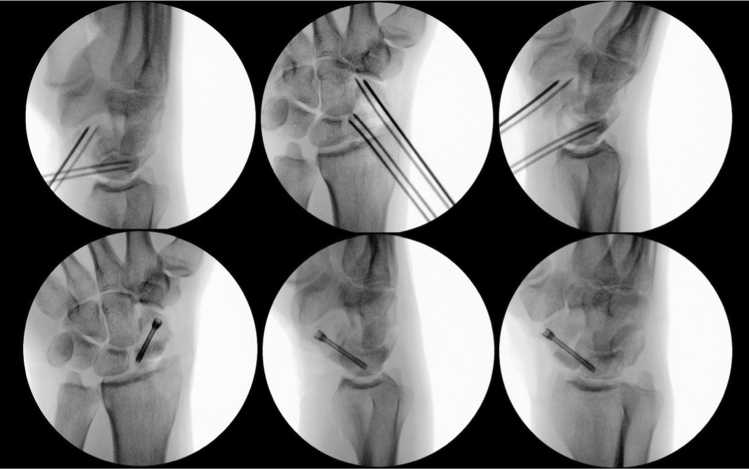
Intraoperative fluoroscopy; upper line: placed K-wires (left); repositioned scaphoid (middle and right), lower line: reconstructed scaphoid with iliac crest bone graft and CCS 3.0 screw

Intraoperative fluoroscopy (Fig. 14) finally confirmed the anatomical reconstruction of the scaphoid nonunion. A cannulated headless screw (CCS 3.0, Medartis AG, Basel, Switzerland) was used for graft fixation.

**Fig. 15:**
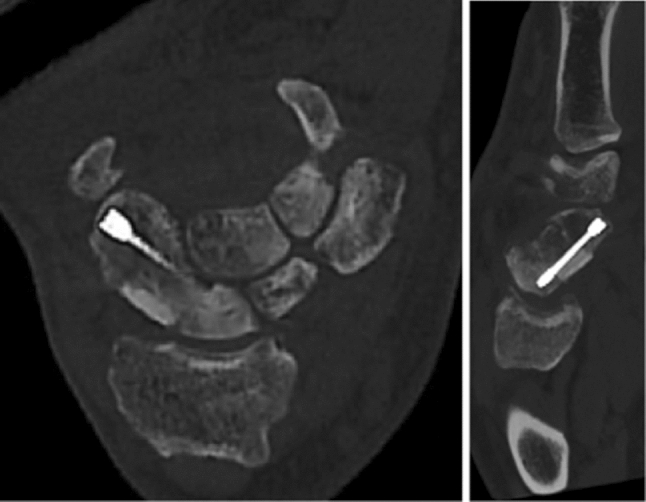
CBCT with Sander's reconstructions 6 weeks postoperatively

The postoperative follow-up after 6 and 12 weeks showed bone union and no radiological signs of loss of reduction (Fig. 15) [19].

**Fig. 16:**
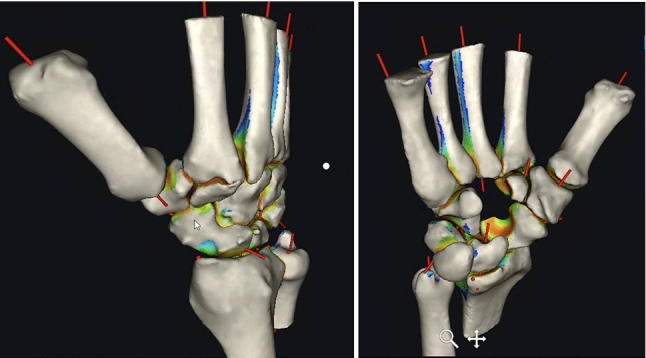
Postoperative analysis with distance mapping A homogenous distribution of the joint surface of the scaphoid (= corresponding joint surface of the removed capitate) indicates an anatomical reconstruction of the scaphoid. (left: radial view, right: ulno-palmar view capitate removed)

For postoperative assessment, three-dimensional models based on the CBCT images were computed using the abovementioned software showing bony union and good alignment of the articular surfaces. The colour maps (orange and red area) in Fig. 16 show a centred position of the scaphoid in the scaphoid fossa as well as a centred position of the capitate in the scapho-capitate articulation and a lunate in a neutral position. The patient reported a VAS of 0 at the 6- and 12-week follow-ups.

## Discussion

The process of generating 3D models starts with high-quality imaging. Depending on the clinical focus and pathology, either MSCT or CBCT can be used. For many carpal pathologies, CBCT is a sufficient method of generating high-quality DICOM data [16, 20].

The most crucial step is segmentation. In a literature review, Van Eijnatten and team showed reported accuracies between 0.04 and 1.9 mm using different segmentation methods [20]. Currently, the main method used in common certified software applications is global thresholding (GT) with accuracies under 0.6 mm, whereas more advanced methods provide accuracy below 0.38 mm. In GT, a fair amount of manual postprocessing is needed, which is a major source of error.

Any further postprocessing, such as computer-aided design (CAD), is based on the results of the segmentation. Further data processing is therefore potentially flawed, leading to unsatisfactory results in additive manufacturing. Errors can be potentiated during further processing of the data, leading to unsatisfactory results of approximately 25% inaccuracy in additive manufacturing reported in the literature [21].

Based on correctly segmented data, distance mapping allows for noninvasive quantification of articular congruency, joint space width, cartilage thickness and interactions of the joints. We used static distance mapping, which was generated by certified software. The visualization of the pre- and postoperative condition in our first case allowed us to simulate the resulting biomechanics of the planned intervention. Based on this information, we changed our surgical plan and avoided a potentially harmful osteotomy of the distal radius and addressed the ulna instead.

Joint congruency of the radio-carpal and mid-carpal joints played a role in our second case. The preoperatively detected, early stage degenerative articular changes could be visualized and compared with the postoperative simulated situation. The findings of Foumani et al. [9], which showed that joint space width can be overestimated in static distance mapping, were considered by planning the intervention using additively manufactured patient-specific guides.

The aim of every scaphoid nonunion reconstruction is not to overstuff the joint but to extend the scaphoid just enough to correct for the adapted DISI (dorsal intercalated segment instability) position of the lunate. Postoperative 3D analysis after 6 weeks using static distance mapping proved the correction of the contact area of the radio-scaphoid joint and an anatomical alignment of the proximal and distal carpal row.

Dynamic distance mapping is currently the most accurate and realistic estimation of the joint space but requires radiological dynamic assessment of wrist motion, such as four-dimensional (4D) CT [9]. Further investigation should focus on more consecutive patients using DM in various pathologies and the integration of 4D CT data with dynamic distance mapping in certified software planning tools to allow simulation of postoperative changes in bone position and contact areas after planned surgical interventions.

Our results are limited due to individual cases presented and patient-specific tools being used for the treatment. However, it seems that our promising results could have an impact on virtual surgical planning and on avoiding mistakes in surgical planning based on 3D models. More studies are needed to prove the generalizability in more patients and more pathologies.

## Conclusion

The use of colour-coded static or dynamic distance mapping is useful for virtual surgical planning of corrective osteotomies of the hand, wrist and forearm. It also allows confirmation of the correct treatment of the patient and assessment of the follow-up radiological documentation.
